# Perceived shared decision-making (SDM) involvement and its association with patient satisfaction among primary healthcare visitors in Makkah city: a cross-sectional study

**DOI:** 10.3389/fmed.2026.1984378

**Published:** 2026-10-20

**Authors:** Alaa Ahmed Alghamdi, Jehad Helal Alqurashi

**Affiliations:** Department of Preventive Medicine, Ministry of Health, Makkah, Saudi Arabia

**Keywords:** patient satisfaction, patient-centered care, primary healthcare, Saudi Arabia, shared decision-making

## Abstract

**Background:**

Shared decision-making (SDM) is an important component of patient-centered care, but evidence regarding its relationship with patient satisfaction in Saudi primary healthcare remains limited. This study assessed perceived SDM involvement among adults attending primary healthcare centers (PHCCs) in Makkah City and examined its cross-sectional association with patient satisfaction.

**Methods:**

A multicenter cross-sectional study was conducted among adults attending eight PHCCs in Makkah City during 2025. PHCCs were selected using stratified random sampling across four geographical sectors, while participants within selected centers were recruited using convenience sampling. Perceived SDM was measured using the 9-item Shared Decision-Making Questionnaire (SDM-Q-9), and patient satisfaction was assessed using the Patient Satisfaction Questionnaire–Short Form (PSQ-18). Spearman's rank correlation and multivariable linear regression were used to assess the association between SDM and satisfaction. HC3 robust standard errors, PHCC-adjusted regression, and median quantile regression were used as sensitivity analyses.

**Results:**

Of 413 completed questionnaires, 407 eligible participants were included. Mean age was 32.1 ± 11.7 years; 72.2% were female and 88.2% were Saudi nationals. The mean SDM-Q-9 score was 53.99 ± 33.03 and the mean PSQ-18 score was 59.20 ± 9.57. SDM-Q-9 scores were weakly positively correlated with PSQ-18 scores (*ρ* = 0.258, *P* < 0.001). In the adjusted model, each 10-point increase in SDM-Q-9 was associated with a 0.91-point higher PSQ-18 score (95% CI, 0.58–1.23; *P* < 0.001). The association remained consistent after PHCC adjustment (0.86; 95% CI, 0.52–1.21) and median quantile regression (0.87; 95% CI, 0.41–1.33).

**Conclusion:**

Greater perceived involvement in shared decision-making was consistently associated with higher patient satisfaction among adults attending PHCCs in Makkah City. Given the cross-sectional design, these findings indicate an association rather than a causal effect.

## 1 Background

Shared decision-making (SDM) is increasingly recognized as a core component of patient-centered care, particularly when clinical decisions involve more than one reasonable option. It involves a collaborative process in which clinicians and patients exchange information, discuss available alternatives, consider patient values and preferences, and reach decisions together (1, 2). Beyond its ethical importance, SDM may improve patients' knowledge, involvement, and confidence in healthcare decisions, although the magnitude of its effects varies across settings and outcomes (3, 4). Evidence from clinical studies and systematic reviews also suggests that SDM can contribute to greater satisfaction with care and treatment decisions, although the strength and consistency of these effects vary across populations and study designs (5, 6).

Patient satisfaction is a complementary indicator of healthcare quality because it captures patients' perceptions of communication, interpersonal relationships, technical care, accessibility, and other aspects of their healthcare experience (7, 8). Research has shown that better patient experiences are associated with favorable healthcare processes and outcomes, making patient-reported measures particularly relevant to quality improvement (7). In primary care, where repeated clinician–patient interactions frequently involve preventive care, chronic disease management, and treatment decisions, effective communication and meaningful patient participation may be especially important. Importantly, patient satisfaction and SDM represent related but distinct constructs: satisfaction reflects a broader evaluation of the healthcare experience, whereas SDM specifically concerns patients’ involvement in the decision-making process.

The relevance of these issues is particularly important in Saudi Arabia, where primary healthcare has undergone substantial development and quality improvement initiatives. Studies from Saudi primary care settings have identified communication, accessibility, continuity, availability of services, and physician-related factors as important determinants of patient satisfaction (9, 10). A systematic review found that communication and access were among the most frequently examined components of satisfaction, while evidence concerning relational conduct, technical competence, and other aspects of the patient–physician relationship was comparatively limited (9). More recent evidence similarly identified accessibility, communication, technical skills, interpersonal conduct, and service availability as important predictors of satisfaction across Saudi primary healthcare settings (10). Waiting time has also been identified as an important determinant of satisfaction in Saudi primary care (11). These findings highlight that satisfaction in Saudi primary care is multidimensional and may reflect factors extending beyond the decision-making process itself.

Despite increasing emphasis on patient-centered care, evidence specifically examining patients' perceived or experienced involvement in SDM during primary-care encounters, and its relationship with overall patient satisfaction, remains limited in Saudi Arabia**.** A Saudi study found that shared decision-making was the most preferred decision-making style among participants, although preferences varied according to demographic characteristics (12). However, preference for a particular decision-making style does not necessarily indicate the extent to which SDM is experienced during actual healthcare encounters. In addition, qualitative research among healthcare professionals working in Saudi PHCCs identified time pressure, physician shortages, limited treatment options, insufficient decision-support resources, and communication challenges as barriers to effective SDM (13). These findings suggest that SDM may be feasible but inconsistently implemented in routine primary care. Thus, assessing patients' perceived involvement during real-world primary-care encounters may provide complementary evidence to studies examining preferred decision-making styles or clinicians' perspectives.

Accordingly, this study aimed to assess perceived SDM involvement among adults attending PHCCs in Makkah City and to examine its cross-sectional association with patient satisfaction using validated patient-reported measures. Secondary analyses explored demographic and healthcare-related factors associated with perceived SDM and examined whether SDM was associated with specific dimensions of patient satisfaction.

## 2 Methods

### 2.1 Study design and setting

A multicenter cross-sectional study was conducted among adults attending primary healthcare centers (PHCCs) in Makkah City, Saudi Arabia, during 2025. The study was conducted across eight PHCCs selected from four geographical sectors of the city: Al-Awali, Al-Sharaie, Al-Kaakiyah, and Al-Zahir. A multistage sampling strategy was used. In the first stage, two PHCCs were selected from each geographical sector using stratified random sampling from the 42 PHCCs listed within the Makkah Health Cluster. The multicenter design was intended to capture variation across different primary-care facilities rather than relying on a single-center sample. Because participants were recruited within multiple PHCCs, PHCC was retained as an analytical variable and was considered in sensitivity analyses to assess whether systematic differences between participating centers influenced the primary association.

### 2.2 Participants and eligibility criteria

Adults aged 18 years or older who attended one of the selected PHCCs for any medical reason were eligible to participate. Participants were required to be able to complete the questionnaire in either Arabic or English. Individuals younger than 18 years, those with serious or terminal illness, and those unable to complete the questionnaire because of language barriers were excluded. Recruitment was conducted within the participating PHCCs using convenience sampling, with trained research assistants approaching eligible patients in waiting areas and inviting them to participate through a bilingual electronic questionnaire. Because individual participants were recruited using convenience sampling rather than probability sampling, the study was not designed to produce population-representative estimates for all PHCC users in Makkah City.

### 2.3 Sample size and sampling procedure

Because the primary research question concerned the association between perceived shared decision-making and patient satisfaction, the sample-size justification was aligned with the planned multivariable regression analysis rather than relying solely on a population-proportion calculation. A regression-based sample-size assessment was conducted using a two-sided significance level of 0.05, 80% statistical power, a small effect size of Cohen's f^2^ = 0.02, one tested predictor (SDM-Q-9), and 12 predictor parameters in the full regression model. The estimated minimum required sample size was 395 participants. The final analytic sample of 407 participants therefore exceeded the estimated requirement for detecting the prespecified primary association under these assumptions.

The 12 predictor parameters comprised SDM-Q-9 score together with age and the categorical covariates included in the primary model (sex, nationality, residency status in Makkah, area of residence, marital status, educational attainment, and frequency of healthcare visits), with multi-category variables contributing more than one parameter. The effect-size assumption was selected to represent a small association and was used to assess the adequacy of the achieved sample for the primary regression analysis. This calculation was intended to address statistical adequacy for the primary individual-level association and was not interpreted as establishing probability-based representativeness of the Makkah population.

A total of 413 completed questionnaires were obtained. During data verification, six respondents were identified as being younger than 18 years and were therefore excluded according to the predefined eligibility criteria, leaving 407 participants in the final analytic sample.

Because participant recruitment within each selected PHCC was based on convenience sampling, the calculated sample size was used to guide recruitment rather than to imply probability-based individual-level sampling. Accordingly, the findings are interpreted as evidence from the participating primary-care population rather than as directly representative population estimates for all residents of Makkah.

### 2.4 Data collection and study variables

Data were collected using a structured bilingual electronic questionnaire administered in Arabic and English through a QR-code link to a Google Forms survey. Research assistants approached patients in PHCC waiting areas and provided information about the study before questionnaire completion. The questionnaire collected age, sex, nationality, residency status in Makkah, PHCC attended, frequency of healthcare visits, area of residence, marital status, and educational attainment. The participating PHCC was retained as an analytical variable to account for the multicenter structure of the data and to permit center-adjusted sensitivity analyses.

The participant-level covariates included in the primary model were selected *a priori* based on their potential relevance to patients' experiences of shared decision-making and satisfaction and their availability in the study dataset. These variables were included as adjustment factors regardless of their individual statistical significance in the primary model. Clinical and encounter-level characteristics that could potentially influence both perceived SDM and satisfaction, including clinical condition or severity, type of consultation, consultation duration, continuity with the physician, physician characteristics, and whether the encounter involved a clinical decision with multiple reasonable alternatives, were not collected.

The primary exposure was patients' perceived involvement in shared decision-making, assessed using the 9-item Shared Decision-Making Questionnaire (SDM-Q-9) (14). The primary outcome was patient satisfaction, assessed using the Patient Satisfaction Questionnaire Short Form (PSQ-18) (15). The statistical analysis distinguished the prespecified primary association between SDM-Q-9 and PSQ-18 from secondary analyses examining factors associated with perceived SDM and relationships between SDM and individual PSQ-18 domains. The primary analysis was interpreted as an association between two patient-reported constructs rather than as evidence of a causal effect.

### 2.5 Measurement of perceived shared decision-making

Perceived shared decision-making was assessed using the SDM-Q-9, a nine-item instrument designed to measure patients’ perceptions of shared decision-making during the clinical consultation (14, 16). The Arabic validated version was used in the present study (16). Each item is rated on a six-point Likert scale ranging from 0 (completely disagree) to 5 (completely agree), giving a total raw score from 0 to 45. For analysis and interpretation, the raw total was transformed linearly to a 0–100 scale, with higher scores indicating greater perceived involvement in shared decision-making. The transformed score was calculated by dividing the raw total score by 45 and multiplying by 100.

### 2.6 Measurement of patient satisfaction

Patient satisfaction was assessed using the PSQ-18, a validated short-form instrument covering multiple aspects of the patient healthcare experience, including general satisfaction, technical quality, interpersonal manner, communication, financial aspects, time spent with the physician, and accessibility/convenience (15, 17). The Arabic-validated version was used (17). Each item is scored on a five-point Likert scale from 1 to 5. Negatively worded items were reverse-scored before calculation of the total score, producing a possible total score ranging from 18 to 90, with higher scores indicating greater satisfaction. The item-level analyses retained the original wording and response orientation, whereas reverse scoring was applied only when deriving the analytical PSQ-18 scores and subscale measures.

For the domain-specific analyses, the seven PSQ-18 domains were constructed according to the original PSQ-18 scoring structure: general satisfaction (items 3 and 17), technical quality (items 2, 4, 6, and 14), interpersonal manner (items 10 and 11), communication (items 1 and 13), financial aspects (items 5 and 7), time spent with the doctor (items 12 and 15), and accessibility/convenience (items 8, 9, 16, and 18). Domain scores were calculated as the mean of the scored items within each domain. Negatively worded items were reverse-scored before calculation of the total and domain scores so that higher scores consistently represented greater satisfaction. The scoring procedure was checked against the validated Arabic version used in the study and the original PSQ-18 scoring structure before analysis.

### 2.7 Data management and quality control

The raw dataset was reviewed before statistical analysis to verify participant eligibility, variable coding, response ranges, and questionnaire completeness. Six records were excluded solely because the respondents were younger than 18 years. Questionnaire responses were then coded according to the predefined scoring rules for the SDM-Q-9 and PSQ-18. All nine SDM-Q-9 and all 18 PSQ-18 items had complete responses among the 407 participants included in the final analysis. Item-level response categories were checked against the original bilingual responses before numerical scoring to minimize errors arising from differences in Arabic and English response formatting.

The PSQ-18 item allocation, reverse-scoring procedure, and domain construction were verified before calculation of the total and domain-specific scores. No imputation was required because all SDM-Q-9 and PSQ-18 items were complete among participants included in the primary analysis.

### 2.8 Statistical analysis

All analyses were performed using R statistical software version 4.3.3. Continuous variables were summarized using means and standard deviations and, where appropriate, medians and interquartile ranges, while categorical variables were presented as frequencies and percentages. Internal consistency of the SDM-Q-9 and PSQ-18 was assessed using Cronbach's alpha, and PSQ-18 domain scores were calculated as mean item scores. The primary analysis evaluated the association between perceived shared decision-making and patient satisfaction, with PSQ-18 total score specified as the outcome and SDM-Q-9 total score as the main exposure. The unadjusted association was examined using Spearman's rank correlation. Multivariable linear regression was then used to adjust for age, sex, nationality, residency status in Makkah, area of residence, marital status, educational attainment, and frequency of healthcare visits. Age was modeled continuously, and the SDM-Q-9 association was expressed as the change in PSQ-18 score per 10-point increase in SDM-Q-9.

The participant-level covariates were retained in the primary model based on their prespecified relevance to potential differences in perceived SDM and patient satisfaction rather than on statistical significance in univariable analyses. Model diagnostics included assessment of linearity, heteroscedasticity, multicollinearity, and influential observations. Because heteroscedasticity was detected, HC3 heteroscedasticity-consistent standard errors and 95% confidence intervals were used for the primary regression. Because participants were recruited from eight PHCCs, the multicenter structure was explicitly considered in the analysis. HC3 robust standard errors were used in the primary model to provide inference that was robust to heteroscedasticity; these standard errors were not interpreted as directly accounting for within-PHCC correlation. To assess whether systematic differences between participating PHCCs materially influenced the observed association, a PHCC-adjusted sensitivity analysis was performed by incorporating PHCC as a fixed-effect term. This analysis was considered complementary to the primary HC3 model and was used to examine the stability of the association after accounting for between-center differences.

Robustness was assessed using conventional regression, the PHCC-adjusted fixed-effect model, and median quantile regression. The consistency of the effect estimate across these alternative specifications was evaluated in terms of its direction, magnitude, and confidence interval rather than by statistical significance alone. Secondary analyses examined factors associated with SDM-Q-9 and associations between SDM-Q-9 and individual PSQ-18 domains; Benjamini–Hochberg adjustment was applied to the seven domain-specific analyses. These analyses were considered secondary and exploratory relative to the prespecified primary SDM-Q-9–PSQ-18 association and were not used to alter the interpretation of the primary analysis. All tests were two-sided, with *P* < 0.05 considered statistically significant. Given that only eight PHCCs participated, the PHCC-adjusted analysis was interpreted as an assessment of systematic between-center differences rather than as a definitive characterization of the underlying population-level clustering structure.

### 2.9 Ethical considerations

The study was conducted in accordance with the principles of the Declaration of Helsinki and was approved by the Institutional Review Board of the Makkah Al-Mukarramah City Local Research Ethics Committee under the Ministry of Health, Saudi Arabia (IRB No. H-02-K-076-0325-1310). All participants provided informed consent before participation. Data were collected anonymously, and confidentiality was maintained throughout the study. The electronic dataset was stored in password-protected files accessible only to members of the research team.

## 3 Results

### 3.1 Study population and participant characteristics

A total of 413 completed questionnaires were received. Following eligibility assessment, six respondents aged <18 years were excluded, leaving 407 participants in the final analytic sample (Figure 1). The mean age was 32.1 ± 11.7 years, with a median of 28.0 years (IQR, 23.0–40.0). Most participants were aged 18–30 years (58.5%), female (72.2%), and Saudi nationals (88.2%). Most were permanent residents of Makkah (92.9%) and lived in urban areas (90.9%). Regarding marital status, 58.5% were single, 31.4% were married, and 10.1% were widowed or divorced. More than half had a university or postgraduate education (56.0%), while 37.6% had completed high school and 6.4% had education up to secondary level. Approximately half reported visiting healthcare services once (51.4%), compared with 32.9% reporting 2–3 visits and 15.7% reporting four or more visits (Table 1).

**Figure 1:**
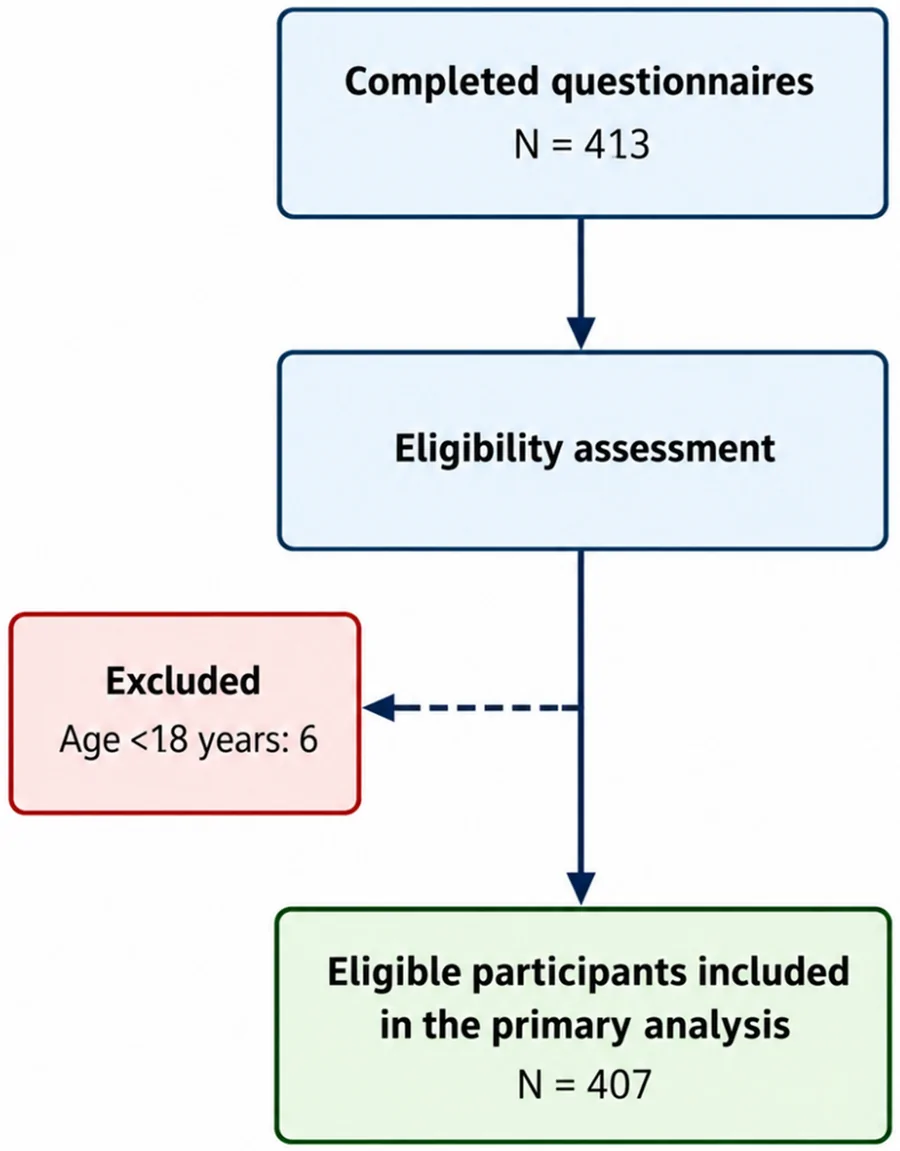
Flow of participants through the study.

**Table 1:** Sociodemographic and healthcare characteristics of the study participants (*N* = 407).

Characteristic	Category	*n* (%)
Age, years [Mean ± SD; median (IQR)]		32.1 ± 11.7; 28.0 [23.0–40.0]
Age group, years	18–30	238 (58.5)
	31–45	113 (27.8)
	≥46	56 (13.8)
Sex	Female	294 (72.2)
	Male	113 (27.8)
Nationality	Saudi	359 (88.2)
	Non-Saudi	48 (11.8)
Residency status in Makkah	Permanent resident	378 (92.9)
	Temporary resident	29 (7.1)
Area of residence	Urban	370 (90.9)
	Rural	37 (9.1)
Marital status	Married	128 (31.4)
	Single	238 (58.5)
	Widowed/divorced	41 (10.1)
Educational attainment	Up to secondary	26 (6.4)
	High school	153 (37.6)
	University/postgraduate	228 (56.0)
Frequency of healthcare visits	Once	209 (51.4)
	2–3 times	134 (32.9)
	≥4 times	64 (15.7)
Primary healthcare center	Al-Aziziyah	83 (20.4)
	Al-Kaakiyah	34 (8.4)
	Al-Nawariyah	47 (11.5)
	Al-Rashidiyah	38 (9.3)
	Al-Sharayaa 7	102 (25.1)
	Al-Zahir	27 (6.6)
	Batha Quraish	37 (9.1)
	Kudai & Al-Hijrah	39 (9.6)

Values are presented as *n* (%) unless otherwise indicated. Age is presented as mean ± standard deviation (SD) and median [interquartile range (IQR)]. Percentages are calculated using the number of non-missing observations for each characteristic. Six respondents younger than 18 years were excluded before the primary analysis, resulting in an analytic sample of 407 participants. The full PHCC distribution is shown to describe the multicenter sampling structure and the distribution of participants across the participating centers.

Participants were recruited from eight PHCCs, with the number of participants ranging from 27 at Al-Zahir to 102 at Al-Sharayaa 7. The demographic composition varied across centers. The proportion of women ranged from 54.2% to 89.7%, while the proportion of Saudi participants ranged from 72.3% to 100.0%. Rural residence ranged from 0% in Al-Kaakiyah and Batha Quraish to 19.3% in Al-Aziziyah. Differences across centers were also observed in marital status, educational attainment, and frequency of healthcare visits (Supplementary Table S1).

### 3.2 SDM-Q-9 and PSQ-18 scores

The mean SDM-Q-9 score was 53.99 ± 33.03 on the transformed 0–100 scale, with a median of 55.56 (IQR, 22.22–82.22) and a range of 0–100. The mean PSQ-18 score was 59.20 ± 9.57, with a median of 57.00 (IQR, 54.00–64.00) and a range of 18–90 (Table 2). Internal consistency was high for the SDM-Q-9 (Cronbach's *α* = 0.970) and acceptable for the PSQ-18 (Cronbach's *α* = 0.728). Among the PSQ-18 domains, communication had the highest mean score (3.59 ± 0.90), followed by general satisfaction (3.56 ± 0.94), whereas time spent with the doctor had the lowest mean score (2.94 ± 0.70). The corresponding means for technical quality, interpersonal manner, financial aspects, and accessibility/convenience were 3.34, 3.08, 3.48, and 3.14, respectively.

**Table 2:** Distribution and internal consistency of SDM-Q-9 and PSQ-18 scores.

Measure	Mean ± SD	Median [IQR]	Range	Cronbach's *α*
SDM-Q-9 total score (0–100)	53.99 ± 33.03	55.56 [22.22–82.22]	0–100	0.970
PSQ-18 total score (18–90)	59.20 ± 9.57	57.00 [54.00–64.00]	18–90	0.728
General satisfaction	3.56 ± 0.94	3.50 [3.00–4.50]	1–5	—
Technical quality	3.34 ± 0.67	3.25 [3.00–3.75]	1–5	—
Interpersonal manner	3.08 ± 0.47	3.00 [3.00–3.00]	1–5	—
Communication	3.59 ± 0.90	3.50 [3.00–4.50]	1–5	—
Financial aspects	3.48 ± 0.88	3.00 [3.00–4.00]	1–5	—
Time spent with doctor	2.94 ± 0.70	3.00 [2.50–3.00]	1–5	—
Accessibility/convenience	3.14 ± 0.70	3.00 [2.75–3.50]	1–5	—

SDM-Q-9 scores were transformed from the original 0–45 score to a 0–100 scale, with higher scores indicating greater perceived involvement in shared decision-making. PSQ-18 total scores range from 18 to 90, with higher scores indicating greater patient satisfaction. Negatively worded PSQ-18 items were reverse-scored before calculation of total and domain scores. The seven PSQ-18 domains were constructed according to the original scoring structure: general satisfaction (items 3 and 17), technical quality (items 2, 4, 6, and 14), interpersonal manner (items 10 and 11), communication (items 1 and 13), financial aspects (items 5 and 7), time spent with doctor (items 12 and 15), and accessibility/convenience (items 8, 9, 16, and 18). Domain scores represent the mean of the scored items within each domain. Cronbach's α is reported for the overall SDM-Q-9 and PSQ-18 instruments; subscale reliability coefficients are not reported because several domains contain only two items and produced unstable or uninterpretable internal-consistency estimates.

At the individual-item level, SDM-Q-9 responses showed considerable variation across the nine items. The proportion of participants selecting either “strongly agree” or “completely agree” was highest for the statement that the doctor helped the patient understand all the information and lower for items involving more active joint evaluation of treatment alternatives (Supplementary Table S2).

For PSQ-18, favorable responses were particularly common for explanations of medical tests and interpersonal behavior. Specifically, 47.2% strongly agreed that doctors were good at explaining the reason for medical tests, while 42.3% strongly agreed that their doctor treated them in a friendly and polite manner. Conversely, access and service-process concerns were evident, with 27.5% strongly agreeing that it was difficult to arrange an appointment and 24.8% strongly agreeing that emergency treatment required excessive waiting. The complete item-level distribution is provided in Supplementary Table S3.

### 3.3 Unadjusted association between perceived shared decision-making and patient satisfaction

The unadjusted analysis demonstrated a weak positive association between perceived shared decision-making and patient satisfaction, with a Spearman correlation coefficient of *ρ* = 0.258 (*P* < 0.001). Thus, higher SDM-Q-9 scores were associated with higher PSQ-18 scores before adjustment for participant characteristics.

### 3.4 Primary multivariable analysis

In the primary multivariable linear regression model, perceived shared decision-making remained positively associated with patient satisfaction after adjustment for age, sex, nationality, residency status, area of residence, marital status, educational attainment, and frequency of healthcare visits (Table 3). For every 10-point increase in SDM-Q-9 score, the adjusted PSQ-18 score was 0.91 points higher (*β* = 0.91, 95% CI, 0.58–1.23; *P* < 0.001).

**Table 3:** Multivariable association between perceived shared decision-making and patient satisfaction: change in PSQ-18 total score per 10-point increase in SDM-Q-9.

Predictor	*β* (95% CI)	Robust SE	*P* value
SDM-Q-9, per 10-point increase	0.91 (0.58 to 1.23)	0.167	<0.001
Age, per 1-year increase	0.06 (−0.04 to 0.16)	0.051	0.255
Male vs. female	−0.25 (−2.64 to 2.15)	1.218	0.839
Saudi vs. non-Saudi	1.50 (−1.43 to 4.42)	1.486	0.315
Temporary vs. permanent resident	2.86 (−1.24 to 6.96)	2.084	0.170
Rural vs. urban residence	−0.83 (−3.85 to 2.19)	1.536	0.588
Single vs. married	1.05 (−1.39 to 3.48)	1.237	0.398
Widowed/divorced vs. married	−2.78 (−6.32 to 0.75)	1.797	0.122
High school vs. university/postgraduate	0.95 (−0.99 to 2.90)	0.989	0.335
Up to secondary vs. university/postgraduate	1.52 (−2.56 to 5.60)	2.075	0.465
≥4 visits vs. 2–3 visits	−1.00 (−4.18 to 2.17)	1.615	0.535
Once vs. 2–3 visits	0.43 (−1.63 to 2.49)	1.048	0.681

β represents the unstandardized regression coefficient. The primary exposure, SDM-Q-9, is expressed per 10-point increase; all other continuous coefficients are expressed per one-unit increase. The outcome was total PSQ-18 score. Models were adjusted for age, sex, nationality, residency status, area of residence, marital status, educational attainment, and frequency of healthcare visits. Heteroscedasticity-consistent HC3 robust standard errors were used for statistical inference in the primary model. PHCC was not included as a covariate in the primary model but was incorporated as a fixed effect in a separate sensitivity analysis (Table 4) to assess whether systematic differences between participating healthcare centers materially influenced the association. Reference categories were female, non-Saudi, permanent resident, urban residence, married, university/postgraduate education, and 2–3 healthcare visits. *N* = 407; *R*^2^ = 0.113; adjusted *R*^2^ = 0.086.

None of the adjusted demographic or healthcare-related covariates was significantly associated with PSQ-18 score. Age showed a small positive but nonsignificant association (*β* = 0.06 per year; 95% CI, −0.04 to 0.16; *P* = 0.255). Similarly, no significant associations were observed for sex, nationality, residency status, area of residence, marital status, education, or healthcare-visit frequency. The largest negative estimate among the covariates was observed for widowed/divorced participants compared with married participants, although this did not reach statistical significance (*β* = −2.78; 95% CI, −6.32 to 0.75; *P* = 0.122).

The primary model explained 11.3% of the variance in PSQ-18 scores (*R*^2^ = 0.113; adjusted *R*^2^ = 0.086). The covariate-adjusted association between SDM-Q-9 and PSQ-18 is illustrated in Figure 2, with the fitted line showing a positive gradient in predicted mean PSQ-18 score across increasing SDM-Q-9 scores.

**Figure 2:**
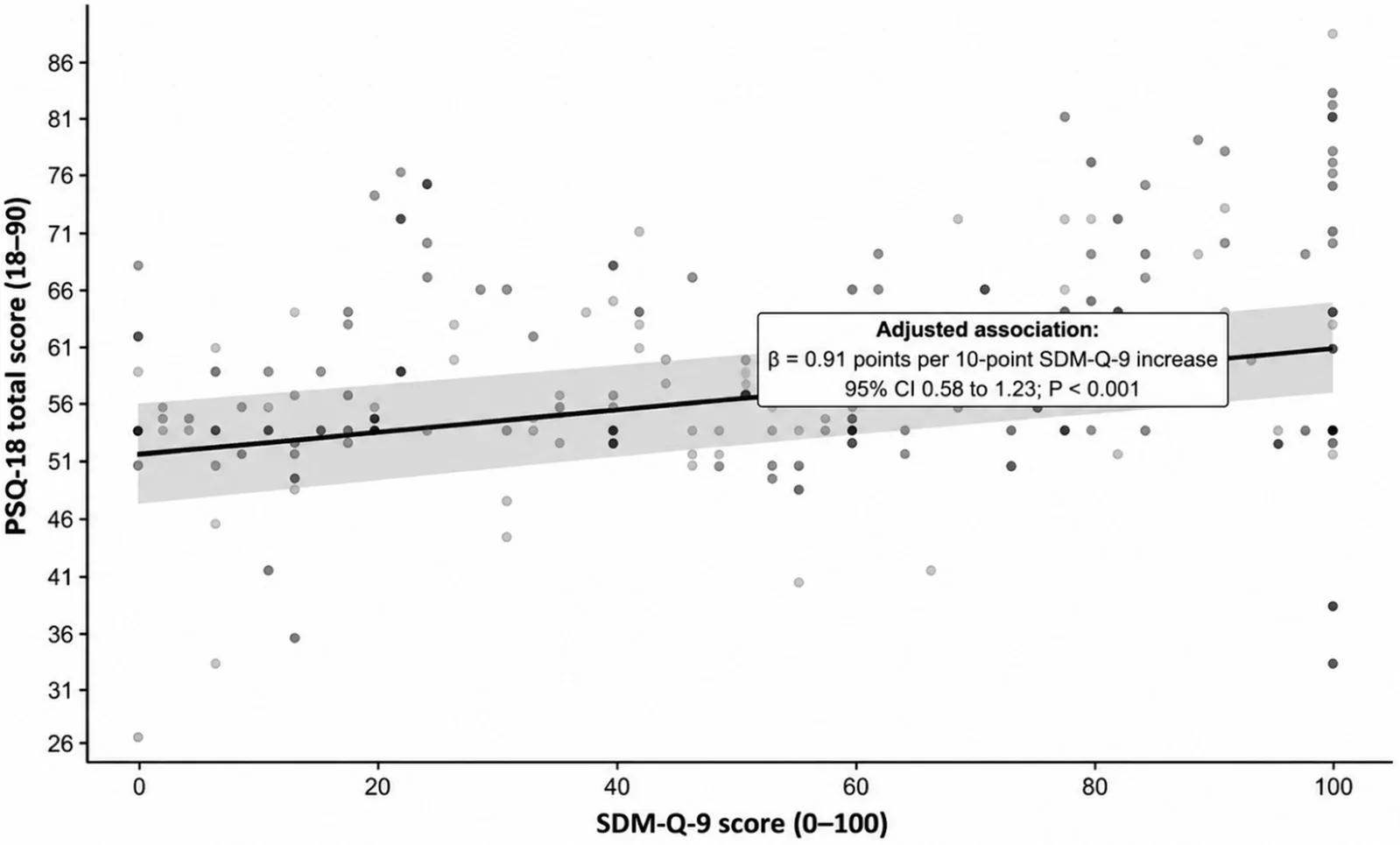
Association between perceived shared decision-making and patient satisfaction. The solid line represents the covariate-adjusted predicted mean PSQ-18 score across the SDM-Q-9 scale, and the shaded area represents the corresponding 95% confidence interval. The displayed estimate represents the change in PSQ-18 total score associated with a 10-point increase in SDM-Q-9.

### 3.5 Robustness and sensitivity analyses

The association between perceived shared decision-making and patient satisfaction was consistent across alternative analytical specifications (Table 4). The conventional multivariable linear regression yielded an estimated difference of 0.91 PSQ-18 points per 10-point increase in SDM-Q-9 (95% CI, 0.63–1.19; *P* < 0.001), compared with 0.91 points (95% CI, 0.58–1.23; *P* < 0.001) using HC3 robust standard errors. After additionally accounting for systematic differences between participating PHCCs using fixed effects, the estimate was 0.86 points (95% CI, 0.52–1.21; *P* < 0.001). Median quantile regression produced a similar estimate of 0.87 points (95% CI, 0.41–1.33; *P* < 0.001). Overall, the direction and magnitude of the association were similar across the alternative models, including the PHCC-adjusted sensitivity analysis.

**Table 4:** Robustness of the association between perceived shared decision-making and patient satisfaction: change in PSQ-18 total score per 10-point increase in SDM-Q-9.

Analytical specification	Change in PSQ-18 total score per 10-point increase in SDM-Q-9	95% CI	*P* value
Unadjusted Spearman correlation	*ρ* = 0.258	—	<0.001
Conventional multivariable linear regression	0.91	0.63 to 1.19	<0.001
HC3 robust multivariable linear regression (primary model)	0.91	0.58 to 1.23	<0.001
PHCC-adjusted linear regression + HC3	0.86	0.52 to 1.21	<0.001
Median quantile regression	0.87	0.41 to 1.33	<0.001

Regression estimates represent the change in PSQ-18 total score associated with a 10-point increase in SDM-Q-9. The Spearman coefficient represents the unadjusted rank-based correlation and is not directly comparable in magnitude with the regression coefficients. The conventional linear model uses ordinary standard errors, whereas the primary model uses HC3 heteroscedasticity-consistent standard errors. The PHCC-adjusted model additionally included PHCC as a fixed-effect term to assess whether systematic differences between participating primary healthcare centers materially influenced the association. The PHCC fixed-effect analysis was considered a sensitivity analysis and was not interpreted as directly modeling residual within-PHCC correlation. Median quantile regression estimates represent the change in the conditional median PSQ-18 score. All adjusted models included the same participant-level covariates as the primary analysis.

### 3.6 Secondary factors associated with perceived shared decision-making

In secondary exploratory analyses, rural residence was associated with higher perceived SDM involvement. Rural participants had SDM-Q-9 scores approximately 13 points higher than urban participants (*β* = 13.09; 95% CI, 3.67–22.51; *P* = 0.007). Saudi nationality was associated with a lower SDM-Q-9 score compared with non-Saudi nationality (*β* = −8.41; 95% CI, −16.56 to −0.25; *P* = 0.043), while single participants had lower scores than married participants (*β* = −9.04; 95% CI, −16.96 to −1.12; *P* = 0.025). Age, residency status, widowed/divorced status, education, and visit frequency were not statistically significant. These subgroup findings should be interpreted cautiously and considered hypothesis-generating, particularly because the rural subgroup included only 37 participants and the non-Saudi subgroup included 48 participants. The model explained 6.4% of the variance in SDM-Q-9 scores (*R*^2^ = 0.064; adjusted *R*^2^ = 0.038). Full estimates are presented in Supplementary Table S4.

### 3.7 Associations with specific dimensions of patient satisfaction

In secondary analyses of the individual PSQ-18 domains, higher SDM-Q-9 scores were positively associated with general satisfaction, technical quality, communication, financial aspects, time spent with the doctor, and accessibility/convenience after adjustment for the same participant-level covariates. For every 10-point increase in SDM-Q-9, the corresponding adjusted differences were 0.07 points for general satisfaction, 0.06 for technical quality, 0.08 for communication, 0.05 for financial aspects, 0.03 for time spent with the doctor, and 0.05 for accessibility/convenience; all remained statistically significant after Benjamini–Hochberg false-discovery-rate correction. In contrast, the association with interpersonal manner was not statistically significant (*β* = 0.01; 95% CI, approximately −0.00 to 0.02; *P* = 0.212; FDR-adjusted *P* = 0.212). These domain-specific findings were considered supplementary to the prespecified primary SDM-Q-9–PSQ-18 association. The complete domain-specific estimates are presented in Supplementary Table S5.

## 4 Discussion

This study examined perceived shared decision-making (SDM) and its relationship with patient satisfaction among adults attending eight primary healthcare centers in Makkah, Saudi Arabia. The main finding was a weak but consistent positive association between perceived SDM involvement and patient satisfaction. The unadjusted association was statistically significant (Spearman's *ρ* = 0.258, *P* < 0.001), and the relationship remained evident after multivariable adjustment and across several sensitivity analyses. Specifically, each 10-point increase in SDM-Q-9 score was associated with a 0.91-point higher PSQ-18 score in the primary HC3-adjusted model, with similar estimates after accounting for PHCC differences and using median quantile regression. The similarity of the estimates across these specifications indicates that the observed association was not materially altered by the choice of regression approach or by adjustment for systematic differences between the participating PHCCs. However, because SDM and satisfaction were measured during the same period in a cross-sectional study, these findings should be interpreted as an association rather than evidence of a causal relationship.

The mean SDM-Q-9 score of 53.99/100 indicates a moderate level of perceived involvement in shared decision-making. This falls within the broad range of SDM-Q-9 means reported across previous studies, where scores have varied considerably according to clinical setting, population, and intervention (18). The result therefore suggests that SDM is occurring to some extent in routine primary care in Makkah, but that patient involvement is not uniformly experienced across all components of the decision-making process. The item-level findings reinforce this interpretation. Participants were relatively more likely to report that physicians helped them understand information, whereas active collaborative behaviors such as thoroughly weighing treatment alternatives together appeared less consistently experienced. This distinction is important because SDM is not simply the provision of information; it encompasses information exchange, discussion of alternatives, consideration of preferences, and agreement on a course of action (1, 2). The observed pattern is also consistent with evidence that patient-reported SDM can differ from objectively observed decision-making behavior, emphasizing that perceived involvement and the actual quality of SDM are related but distinct constructs (19).

The positive relationship between SDM and satisfaction is consistent with the broader literature. A systematic review of randomized trials found mixed but generally favorable evidence regarding the effects of SDM on patient outcomes, including satisfaction (5). Similarly, a meta-analysis found that involvement in treatment choices or receiving preferred treatment was associated with higher treatment satisfaction, although the average effects were modest (6). Evidence from primary care also suggests that patients' experience of the decision-making process is related to their satisfaction and trust. In a direct observation study, discordance between patients’ desired and experienced decision-making was associated with lower satisfaction and trust (19). More recently, a large cross-sectional study among 12,401 outpatients reported that factors such as communication quality and consultation duration were associated with satisfaction with SDM involvement, with trust emerging as an important explanatory factor (20). These findings are broadly compatible with our results, although differences in populations, SDM measures, outcomes, and study designs make direct comparison of effect sizes inappropriate. Our findings extend this literature by demonstrating a similar positive relationship in routine primary healthcare in Saudi Arabia using a patient-reported measure of perceived SDM.

The magnitude of the association in the present study should nevertheless be interpreted as modest rather than large. A 10-point increase in SDM-Q-9 corresponded to approximately 0.9 points on the 18–90 PSQ-18 scale. This is clinically plausible because patient satisfaction is multifactorial and is influenced by many aspects of the healthcare encounter beyond decision-making, including access, waiting time, continuity, technical competence, communication, and organizational factors. The relatively modest model *R*^2^ further supports this interpretation, as most variation in satisfaction remained unexplained by the variables included in the current model (21). Rather than suggesting that SDM alone determines satisfaction, the findings support the interpretation that SDM may represent one modifiable component of a broader patient experience (21). Importantly, statistical significance should not be equated with clinical or practical significance. A minimal clinically important difference for the total PSQ-18 score has not been established in the present study, and therefore the 0.91-point difference per 10-point SDM-Q-9 increase should not be interpreted as demonstrating a clinically meaningful improvement in satisfaction.

This interpretation is particularly relevant to the Saudi primary-care context. Previous Saudi studies have identified communication, accessibility, availability of services, interpersonal conduct, and technical aspects of care as important dimensions of patient satisfaction (9, 10). A systematic review of Saudi primary-care studies found that most research focused on availability, accessibility, and communication, while fewer studies examined relational conduct and patients' perceptions of physicians' technical skills (9). A more recent systematic review similarly identified availability, accessibility, communication, interpersonal conduct, technical skills, and personal qualities as recurring predictors of satisfaction (10). Our domain-specific findings are consistent with this multidimensional understanding: higher SDM-Q-9 scores were associated with greater general satisfaction, technical quality, communication, financial aspects, time spent with the doctor, and accessibility/convenience. The strongest domain-specific association was observed for communication, supporting the conceptual link between meaningful patient involvement and effective clinician–patient communication (22). These domain-specific findings should nevertheless be regarded as secondary evidence and should not be interpreted as demonstrating separate causal effects of SDM on each aspect of satisfaction.

The lack of a significant association between SDM-Q-9 and the interpersonal-manner domain is also informative. SDM involves more than being courteous or friendly; it requires specific communication behaviors related to deliberation, preference elicitation, and agreement about decisions (1, 2). A physician may therefore demonstrate good interpersonal manners without necessarily engaging the patient deeply in clinical decision-making. Conversely, SDM may improve patients' perceptions of communication and participation without substantially changing their assessment of interpersonal politeness. This distinction is consistent with research showing that SDM and broader patient-centered communication are related but overlapping constructs rather than interchangeable concepts (23). Thus, the present findings support maintaining a conceptual distinction between interpersonal quality and decision-making involvement.

The secondary analysis identified several factors associated with perceived SDM. Rural participants had substantially higher SDM-Q-9 scores than urban participants after adjustment. This finding should be interpreted cautiously because the rural subgroup included only 37 participants, making the estimate relatively imprecise and potentially sensitive to the characteristics of a small subgroup. Previous Saudi research has demonstrated that preferences for participation in decision-making vary according to sociodemographic characteristics, and that older age, sex, and education can influence preferred decision-making style (12). Differences between rural and urban participants in the present study could reflect variation in physician–patient continuity, consultation patterns, community characteristics, or expectations of primary care (24). However, the available data do not allow these mechanisms to be established. Accordingly, the rural finding should be considered hypothesis-generating rather than evidence that rural care inherently provides better SDM.

Saudi nationality and single marital status were also associated with lower perceived SDM scores in the secondary model. These findings should similarly be regarded as exploratory rather than definitive, particularly because only 48 participants were non-Saudi and subgroup estimates may therefore be unstable. These findings differ in some respects from earlier Saudi research. In a study of 236 patients in Riyadh, shared decision-making was the most preferred decision-making style (57%), while preference for participation was higher among men and participants with higher educational attainment; older participants were more likely to prefer a paternalistic approach (12). Importantly, that study assessed patients' preferred decision-making style, whereas the present study assessed patients' perceived or experienced involvement during healthcare encounters. These constructs should not be assumed to be equivalent. Cultural expectations, previous healthcare experiences, communication patterns, and the clinical context may influence these two constructs differently.

The secondary education findings require particularly cautious interpretation. In the previous analysis, using the small up-to-secondary group as the reference produced apparently large differences across education categories. In the revised analysis, university/postgraduate education was used as the reference because it was the largest group, and education was no longer significantly associated with SDM-Q-9 score. The revised analysis therefore does not provide evidence of a statistically significant association between educational attainment and perceived SDM in this sample. This change illustrates the importance of considering category size and reference-group selection when interpreting subgroup associations (25). It also reduces the likelihood that the earlier counterintuitive education findings were being driven primarily by an unstable comparison with a very small reference category.

The findings also have implications for SDM implementation in Saudi primary care. Qualitative research among healthcare professionals in Saudi PHCCs identified time pressure, physician shortages, limited treatment options, limited decision-support resources, and communication difficulties as barriers to effective SDM (13). These barriers provide a plausible context for the moderate SDM score observed in the present study and for the weaker performance of items requiring active deliberation and joint selection of treatment. Importantly, SDM should not be conceptualized as an additional isolated task imposed on already time-constrained clinicians. Rather, effective SDM can be incorporated into routine consultation processes through structured communication, explicit presentation of options, preference elicitation, and confirmation of agreement. Reviews of SDM interventions in primary care indicate that decision aids can improve knowledge, reduce decisional conflict, and increase satisfaction with decisions, although implementation remains context dependent (26). The present cross-sectional findings do not establish that implementing these approaches would increase satisfaction in this setting; rather, they provide a rationale for evaluating such interventions prospectively in Saudi primary care.

The present results also suggest that improvement efforts should focus on the quality of the decision-making conversation, rather than satisfaction alone. Previous research has shown that global satisfaction may remain high even when specific elements of the decision-making process are suboptimal (27). This is relevant because our participants generally reported favorable perceptions of some aspects of their healthcare experience while demonstrating less consistent involvement in deliberative aspects of decision-making. Patient satisfaction therefore should not be treated as a substitute measure for SDM. Instead, SDM-Q-9 and PSQ-18 appear to capture related but distinct dimensions of the patient experience, and their joint use may provide a more informative assessment of patient-centered primary care.

An additional consideration is residual confounding. Although the primary model adjusted for several demographic and healthcare-utilization characteristics, potentially important clinical and encounter-level factors were not measured. These included clinical condition or severity, type of consultation, consultation duration, continuity with the physician, physician characteristics, and whether the encounter involved a clinical decision among multiple reasonable alternatives. Such factors may influence both perceived SDM and satisfaction and could therefore partly explain the observed association. Reverse directionality is also plausible: patients who were more satisfied with their overall encounter may have been more likely to perceive or report greater involvement in decision-making. Because both constructs were self-reported and assessed during the same period, common underlying factors or reciprocal reporting may also have contributed to the observed relationship.

Finally, the stability of the SDM–satisfaction association across the analytical approaches strengthens the consistency of the central finding. The association remained positive with conventional linear regression, HC3 robust regression, PHCC-adjusted analysis, and median quantile regression. This is particularly relevant given the multicenter structure of the study and the heteroscedasticity identified in the regression diagnostics. The similar estimate after incorporating PHCC fixed effects suggests that systematic differences between the participating health centers did not materially change the estimated association. However, this sensitivity analysis should not be interpreted as demonstrating that all within-PHCC correlation has been eliminated, particularly given that only eight PHCCs were included. Nevertheless, the findings should be understood as evidence of an association between two patient-reported constructs measured during the same period, rather than evidence that increasing SDM would necessarily cause a corresponding increase in satisfaction.

### 4.1 Limitations

Several limitations should be considered when interpreting these findings. First, the cross-sectional design prevents establishing temporal or causal relationships between perceived shared decision-making and patient satisfaction. Because SDM and satisfaction were assessed during the same healthcare period, reverse directionality is also possible; patients who were generally more satisfied with their encounter may have been more likely to perceive or report greater involvement in decision-making. Thus, the observed association may reflect reciprocal reporting, shared underlying determinants, or other unmeasured factors rather than an independent effect of SDM on satisfaction. Accordingly, the findings should not be interpreted as evidence that increasing SDM will necessarily cause higher patient satisfaction.

Second, although PHCCs were selected using stratified random sampling across four geographical sectors, participants within each center were recruited using convenience sampling, which may introduce selection bias and limits direct generalizability to all primary healthcare users in Makkah or elsewhere. Because the number of eligible patients approached and those who declined participation was not systematically recorded, a formal response rate could not be calculated, and the possibility of non-response bias cannot be excluded. Third, the study relied on self-reported questionnaire responses, making the findings susceptible to recall and social-desirability bias, particularly when evaluating physicians and healthcare services. In addition, because both the exposure and outcome were measured using self-reported instruments during the same period, common-method reporting bias may have contributed to the observed association.

Fourth, the sample was predominantly female and Saudi, while some subgroups, particularly rural and non-Saudi participants, were relatively small; estimates for these groups should therefore be interpreted cautiously. In particular, the rural subgroup comprised 37 participants and the non-Saudi subgroup comprised 48 participants, limiting the precision and stability of subgroup estimates. The observed associations involving rural residence, nationality, and marital status should therefore be regarded as exploratory and hypothesis-generating rather than definitive. Fifth, the models explained only a modest proportion of the variability in patient satisfaction and perceived SDM, indicating that important clinical, relational, organizational, and system-level determinants were not measured. Potentially relevant unmeasured factors included clinical condition or severity, type of consultation, consultation duration, continuity with the physician, physician characteristics, and whether the encounter involved a clinical decision among multiple reasonable alternatives. These factors may influence both perceived SDM and patient satisfaction and could therefore contribute to residual confounding.

Sixth, although PHCC was incorporated as a fixed effect in a sensitivity analysis, this approach was intended to assess whether systematic differences between participating centers materially influenced the association and does not directly model residual within-PHCC correlation. Moreover, only eight PHCCs participated, limiting the precision with which between-center variability can be characterized. The similar estimates observed after PHCC adjustment nevertheless provide evidence that the primary association was not substantially altered by systematic differences across the participating centers. Finally, although several robustness analyses were conducted, residual confounding cannot be excluded. The modest magnitude of the primary association should also be distinguished from clinical or practical significance, as a minimal clinically important difference for the total PSQ-18 score was not established in the present study. Therefore, statistical significance should not be interpreted as demonstrating a clinically meaningful effect.

## 5 Conclusion

In this multicenter cross-sectional study of adults attending primary healthcare centers in Makkah, perceived involvement in shared decision-making was moderate and was consistently associated with higher patient satisfaction. Higher SDM-Q-9 scores remained associated with higher PSQ-18 scores after adjustment for demographic and healthcare-related characteristics, and the direction and magnitude of the association were similar across the heteroscedasticity-robust, PHCC-adjusted, and median quantile regression models. Secondary analyses indicated that perceived SDM was also associated with several specific dimensions of the patient experience, particularly communication and general satisfaction; however, these findings were secondary to the prespecified primary analysis and should be interpreted as supplementary rather than definitive. The modest magnitude of the primary association and the relatively limited explanatory power of the models indicate that patient satisfaction is influenced by multiple factors beyond perceived SDM alone. The findings should not be interpreted as evidence of a causal effect of SDM on satisfaction, particularly given the cross-sectional design, convenience sampling, potential residual confounding, and use of self-reported measures. Longitudinal and intervention-based studies are warranted to determine whether changes in perceived SDM are associated with subsequent changes in patient satisfaction and other patient-reported outcomes.

## Data Availability

The original contributions presented in the study are included in the article/Supplementary Material, further inquiries can be directed to the corresponding author/s.
