# Supplementary material for: Perceived shared decision-making (SDM) involvement and its association with patient satisfaction among primary healthcare visitors in Makkah city: a cross-sectional study
Source: Front Med (Lausanne). 2026 Oct 20;13:1984378. doi: 10.3389/fmed.2026.1984378 (PMC13727600; doi:10.3389/fmed.2026.1984378)
Supplement: Supplementary file 1 [file Supplementaryfile1.docx]

**Table S1.** Participant characteristics by primary healthcare center

| **Characteristic** | **Al-Aziziyah *(N=83)*** | **Al-Kaakiyah *(N=34)*** | **Al-Nawariyah *(N=47)*** | **Al-Rashidiyah *(N=38)*** | **Al-Sharayaa 7 *(N=102)*** | **Al-Zahir *(N=27)*** | **Batha Quraish *(N=37)*** | **Kudai & Al-Hijrah *(N=39)*** |
| --- | --- | --- | --- | --- | --- | --- | --- | --- |
| **Age, years** | 39.7 ± 14.4; median 38.0 [27.5–51.0] | 38.9 ± 13.4; median 41.0 [23.0–50.0] | 30.0 ± 10.6; median 26.0 [22.0–34.0] | 31.4 ± 7.2; median 33.0 [25.0–38.0] | 28.6 ± 9.8; median 25.0 [23.0–30.0] | 32.8 ± 9.6; median 37.0 [24.0–40.0] | 25.3 ± 7.2; median 22.0 [21.0–27.0] | 27.8 ± 6.5; median 26.0 [23.0–30.0] |
| **Sex** |  |  |  |  |  |  |  |  |
| Female | 45 (54.2%) | 29 (85.3%) | 39 (83.0%) | 26 (68.4%) | 71 (69.6%) | 21 (77.8%) | 28 (75.7%) | 35 (89.7%) |
| Male | 38 (45.8%) | 5 (14.7%) | 8 (17.0%) | 12 (31.6%) | 31 (30.4%) | 6 (22.2%) | 9 (24.3%) | 4 (10.3%) |
| **Nationality** |  |  |  |  |  |  |  |  |
| Saudi | 60 (72.3%) | 34 (100.0%) | 43 (91.5%) | 36 (94.7%) | 98 (96.1%) | 26 (96.3%) | 29 (78.4%) | 33 (84.6%) |
| Non-Saudi | 23 (27.7%) | 0 (0.0%) | 4 (8.5%) | 2 (5.3%) | 4 (3.9%) | 1 (3.7%) | 8 (21.6%) | 6 (15.4%) |
| **Residency status in Makkah** |  |  |  |  |  |  |  |  |
| Permanent resident | 72 (86.7%) | 34 (100.0%) | 46 (97.9%) | 34 (89.5%) | 96 (94.1%) | 24 (88.9%) | 33 (89.2%) | 39 (100.0%) |
| Temporary resident | 11 (13.3%) | 0 (0.0%) | 1 (2.1%) | 4 (10.5%) | 6 (5.9%) | 3 (11.1%) | 4 (10.8%) | 0 (0.0%) |
| **Area of residence** |  |  |  |  |  |  |  |  |
| Urban | 67 (80.7%) | 34 (100.0%) | 44 (93.6%) | 33 (86.8%) | 94 (92.2%) | 24 (88.9%) | 37 (100.0%) | 37 (94.9%) |
| Rural | 16 (19.3%) | 0 (0.0%) | 3 (6.4%) | 5 (13.2%) | 8 (7.8%) | 3 (11.1%) | 0 (0.0%) | 2 (5.1%) |
| **Marital status** |  |  |  |  |  |  |  |  |
| Married | 33 (39.8%) | 18 (52.9%) | 14 (29.8%) | 7 (18.4%) | 24 (23.5%) | 8 (29.6%) | 11 (29.7%) | 13 (33.3%) |
| Single | 37 (44.6%) | 13 (38.2%) | 31 (66.0%) | 28 (73.7%) | 63 (61.8%) | 16 (59.3%) | 26 (70.3%) | 24 (61.5%) |
| Widowed/divorced | 13 (15.7%) | 3 (8.8%) | 2 (4.3%) | 3 (7.9%) | 15 (14.7%) | 3 (11.1%) | 0 (0.0%) | 2 (5.1%) |
| **Educational attainment** |  |  |  |  |  |  |  |  |
| Up to secondary | 14 (16.9%) | 6 (17.6%) | 1 (2.1%) | 0 (0.0%) | 4 (3.9%) | 0 (0.0%) | 0 (0.0%) | 1 (2.6%) |
| High school | 24 (28.9%) | 5 (14.7%) | 18 (38.3%) | 22 (57.9%) | 35 (34.3%) | 12 (44.4%) | 20 (54.1%) | 17 (43.6%) |
| University/postgraduate | 45 (54.2%) | 23 (67.6%) | 28 (59.6%) | 16 (42.1%) | 63 (61.8%) | 15 (55.6%) | 17 (45.9%) | 21 (53.8%) |
| **Frequency of healthcare visits** |  |  |  |  |  |  |  |  |
| Once | 28 (33.7%) | 22 (64.7%) | 29 (61.7%) | 23 (60.5%) | 50 (49.0%) | 20 (74.1%) | 21 (56.8%) | 16 (41.0%) |
| 2–3 times | 37 (44.6%) | 7 (20.6%) | 13 (27.7%) | 11 (28.9%) | 39 (38.2%) | 3 (11.1%) | 9 (24.3%) | 15 (38.5%) |
| ≥4 times | 18 (21.7%) | 5 (14.7%) | 5 (10.6%) | 4 (10.5%) | 13 (12.7%) | 4 (14.8%) | 7 (18.9%) | 8 (20.5%) |

**Note:** Values are presented as *n (%)* unless otherwise indicated. Age is presented as mean ± standard deviation (SD) and median [interquartile range (IQR)]. Percentages are calculated within each PHCC. A value of 0 (0.0%) indicates that no participant in that PHCC belonged to the corresponding category. The table describes the distribution of participant characteristics across the eight participating primary healthcare centers and is intended as a supplementary description of the multicenter sample.

**Table S2.** Item-level response distribution of the 9-item Shared Decision-Making Questionnaire (SDM-Q-9)

| **Item** | **Statement** | **N** | **Completely disagree, n (%)** | **Strongly disagree, n (%)** | **Somewhat disagree, n (%)** | **Somewhat agree, n (%)** | **Strongly agree, n (%)** | **Completely agree, n (%)** |
| --- | --- | --- | --- | --- | --- | --- | --- | --- |
| SDM-Q-9 item 1 | 1. My doctor made clear that a decision needs to be made | 407 | 82 (20.1) | 36 (8.8) | 59 (14.5) | 89 (21.9) | 39 (9.6) | 102 (25.1) |
| SDM-Q-9 item 2 | 2. My doctor wanted to know exactly how I want to be involved in making the decision | 407 | 78 (19.2) | 53 (13.0) | 59 (14.5) | 81 (19.9) | 37 (9.1) | 99 (24.3) |
| SDM-Q-9 item 3 | 3. My doctor told me that there are different options for treating my medical condition | 407 | 76 (18.7) | 61 (15.0) | 44 (10.8) | 69 (17.0) | 43 (10.6) | 114 (28.0) |
| SDM-Q-9 item 4 | 4. My doctor precisely explained the advantages and disadvantages of the treatment options | 407 | 59 (14.5) | 66 (16.2) | 50 (12.3) | 71 (17.4) | 59 (14.5) | 102 (25.1) |
| SDM-Q-9 item 5 | 5. My doctor helped me understand all the information | 407 | 44 (10.8) | 75 (18.4) | 45 (11.1) | 51 (12.5) | 69 (17.0) | 123 (30.2) |
| SDM-Q-9 item 6 | 6. My doctor asked me which treatment option I prefer | 407 | 80 (19.7) | 50 (12.3) | 54 (13.3) | 70 (17.2) | 48 (11.8) | 105 (25.8) |
| SDM-Q-9 item 7 | 7. My doctor and I thoroughly weighed the different treatment options | 407 | 70 (17.2) | 81 (19.9) | 62 (15.2) | 52 (12.8) | 57 (14.0) | 85 (20.9) |
| SDM-Q-9 item 8 | 8. My doctor and I selected a treatment option together | 407 | 84 (20.6) | 56 (13.8) | 52 (12.8) | 59 (14.5) | 43 (10.6) | 113 (27.8) |
| SDM-Q-9 item 9 | 9. My doctor and I reached an agreement on how to proceed | 407 | 79 (19.4) | 48 (11.8) | 46 (11.3) | 51 (12.5) | 68 (16.7) | 115 (28.3) |

**Note:** Values are presented as n (%). Responses were classified directly from the original bilingual questionnaire responses before numerical scoring. Each SDM-Q-9 item is scored from 0 (completely disagree) to 5 (completely agree). Percentages are calculated using non-missing responses for each item. N = 407 for all items.

**Table S3.** Item-level response distribution of the Patient Satisfaction Questionnaire–Short Form (PSQ-18)

| **Item** | **Statement** | **N** | **Strongly disagree, n (%)** | **Disagree, n (%)** | **Uncertain, n (%)** | **Agree, n (%)** | **Strongly agree, n (%)** |
| --- | --- | --- | --- | --- | --- | --- | --- |
| PSQ-18 item 1 | 1. Doctors are good about explaining the reason for medical tests | 407 | 0 (0.0) | 0 (0.0) | 34 (8.4) | 181 (44.5) | 192 (47.2) |
| PSQ-18 item 2 | 2. I think my doctor's office has everything needed to provide complete medical care | 407 | 0 (0.0) | 0 (0.0) | 91 (22.4) | 174 (42.8) | 142 (34.9) |
| PSQ-18 item 3 | 3. The medical care provided to me is excellent | 407 | 0 (0.0) | 0 (0.0) | 53 (13.0) | 182 (44.7) | 172 (42.3) |
| PSQ-18 item 4 | 4. Sometimes, my doctor makes me wonder if his diagnosis is correct or not | 407 | 0 (0.0) | 0 (0.0) | 114 (28.0) | 204 (50.1) | 89 (21.9) |
| PSQ-18 item 5 | 5. I feel confident that I can get the medical care I need without financial troubles | 407 | 0 (0.0) | 0 (0.0) | 66 (16.2) | 154 (37.8) | 187 (45.9) |
| PSQ-18 item 6 | 6. When I go for medical care, my doctor is careful to check and treat everything completely | 407 | 0 (0.0) | 0 (0.0) | 71 (17.4) | 172 (42.3) | 164 (40.3) |
| PSQ-18 item 7 | 7. I have to pay for my medical care more than I can afford | 407 | 0 (0.0) | 0 (0.0) | 69 (17.0) | 244 (60.0) | 94 (23.1) |
| PSQ-18 item 8 | 8. I have easy access to medical specialists I need | 407 | 0 (0.0) | 0 (0.0) | 84 (20.6) | 198 (48.6) | 125 (30.7) |
| PSQ-18 item 9 | 9. I have to wait too long for emergency treatment | 407 | 0 (0.0) | 0 (0.0) | 86 (21.1) | 220 (54.1) | 101 (24.8) |
| PSQ-18 item 10 | 10. My doctor treats me in an efficient way, not in a personal way | 407 | 0 (0.0) | 0 (0.0) | 64 (15.7) | 196 (48.2) | 147 (36.1) |
| PSQ-18 item 11 | 11. My doctor treats me friendly and politely | 407 | 0 (0.0) | 0 (0.0) | 57 (14.0) | 178 (43.7) | 172 (42.3) |
| PSQ-18 item 12 | 12. Sometimes my doctor examines and diagnoses quickly | 407 | 0 (0.0) | 0 (0.0) | 95 (23.3) | 225 (55.3) | 87 (21.4) |
| PSQ-18 item 13 | 13. Sometimes my doctor ignores what I tell him | 407 | 0 (0.0) | 0 (0.0) | 75 (18.4) | 253 (62.2) | 79 (19.4) |
| PSQ-18 item 14 | 14. I have some doubts about my doctor's medical abilities | 407 | 0 (0.0) | 0 (0.0) | 110 (27.0) | 224 (55.0) | 73 (17.9) |
| PSQ-18 item 15 | 15. My doctor spends too long time with me | 407 | 0 (0.0) | 0 (0.0) | 90 (22.1) | 229 (56.3) | 88 (21.6) |
| PSQ-18 item 16 | 16. I find it difficult to set a date for a check with my doctor | 407 | 0 (0.0) | 0 (0.0) | 92 (22.6) | 203 (49.9) | 112 (27.5) |
| PSQ-18 item 17 | 17. I am not satisfied with the service provided by my doctor | 407 | 0 (0.0) | 0 (0.0) | 73 (17.9) | 252 (61.9) | 82 (20.1) |
| PSQ-18 item 18 | 18. I can get doctor service at any time | 407 | 0 (0.0) | 0 (0.0) | 75 (18.4) | 196 (48.2) | 136 (33.4) |

**Note:** Values are presented as n (%). Responses were classified directly from the original bilingual questionnaire responses before numerical scoring or reverse scoring. The table preserves the original wording and response orientation of each PSQ-18 item. Negatively worded items were reverse-scored only when calculating the PSQ-18 summary score. Percentages are calculated using non-missing responses for each item. N = 407 for all items.

**Table S4.** Secondary multivariable analysis of factors associated with perceived shared decision-making

Outcome: SDM-Q-9 total score (0–100)

| **Predictor** | **β (95% CI)** | **Robust SE** | **P value** |
| --- | --- | --- | --- |
| Age, per 1-year increase | −0.26 (−0.58 to 0.05) | 0.160 | 0.099 |
| Male vs female | 0.42 (−7.25 to 8.10) | 3.906 | 0.913 |
| Saudi vs non-Saudi | −8.41 (−16.56 to −0.25) | 4.148 | 0.043 |
| Temporary vs permanent resident | −2.81 (−14.27 to 8.65) | 5.829 | 0.630 |
| Rural vs urban residence | **13.09 (3.67 to 22.51)** | 4.789 | **0.007** |
| Single vs married | −9.04 (−16.96 to −1.12) | 4.029 | 0.025 |
| Widowed/divorced vs married | −2.11 (−14.03 to 9.82) | 6.067 | 0.729 |
| High school vs university/postgraduate | −4.03 (−10.82 to 2.75) | 3.451 | 0.243 |
| Up to secondary vs university/postgraduate | 10.48 (−1.83 to 22.79) | 6.260 | 0.095 |
| ≥4 visits vs 2–3 visits | 3.22 (−7.12 to 13.56) | 5.259 | 0.541 |
| Once vs 2–3 visits | −3.99 (−11.16 to 3.17) | 3.643 | 0.274 |

**Note:** β represents the unstandardized regression coefficient. The outcome was the SDM-Q-9 total score transformed to a 0–100 scale. Models were adjusted for age, sex, nationality, residency status, area of residence, marital status, educational attainment, and frequency of healthcare visits. HC3 heteroscedasticity-consistent robust standard errors were used. This analysis was considered secondary/exploratory. Reference categories were female, non-Saudi, permanent resident, urban residence, married, university/postgraduate education, and 2–3 healthcare visits. Model R² = 0.064; adjusted R² = 0.038.

**Table S5.** Adjusted associations between perceived shared decision-making and dimensions of patient satisfaction

| **PSQ-18 domain** | **β (95% CI)** | **Robust SE** | **P value** | **FDR-adjusted P** |
| --- | --- | --- | --- | --- |
| **General satisfaction** | **0.07 (0.04 to 0.10)** | 0.015 | <0.001 | <0.001 |
| **Technical quality** | **0.06 (0.04 to 0.08)** | 0.012 | <0.001 | <0.001 |
| Interpersonal manner | 0.01 (−0.00 to 0.02) | 0.007 | 0.212 | 0.212 |
| **Communication** | **0.08 (0.05 to 0.11)** | 0.015 | <0.001 | <0.001 |
| **Financial aspects** | **0.05 (0.02 to 0.08)** | 0.014 | <0.001 | <0.001 |
| **Time spent with doctor** | **0.03 (0.01 to 0.06)** | 0.012 | 0.006 | 0.007 |
| **Accessibility/convenience** | **0.05 (0.02 to 0.07)** | 0.011 | <0.001 | <0.001 |

**Note:** Each PSQ-18 domain was modeled separately using multivariable linear regression with adjustment for age, sex, nationality, residency status, area of residence, marital status, educational attainment, and frequency of healthcare visits. Estimates represent the expected change in subscale score associated with a 10-point increase in SDM-Q-9. HC3 heteroscedasticity-consistent robust standard errors were used. P values were adjusted across the seven secondary domain-specific analyses using the Benjamini–Hochberg false discovery rate procedure. These analyses were considered secondary and should not be interpreted as independent confirmatory hypotheses.
